# Most Networks in Wagner's Model Are Cycling

**DOI:** 10.1371/journal.pone.0034285

**Published:** 2012-04-12

**Authors:** Ricardo Pinho, Elhanan Borenstein, Marcus W. Feldman

**Affiliations:** 1 Department of Biology, Stanford University, Stanford, California, United States of America; 2 PhD Program in Computational Biology, Instituto Gulbenkian de Ciência, Oeiras, Portugal; 3 Department of Genome Sciences, University of Washington, Seattle, Washington, United States of America; University of Maribor, Slovenia

## Abstract

In this paper we study a model of gene networks introduced by Andreas Wagner in the 1990s that has been used extensively to study the evolution of mutational robustness. We investigate a range of model features and parameters and evaluate the extent to which they influence the probability that a random gene network will produce a fixed point steady state expression pattern. There are many different types of models used in the literature, (discrete/continuous, sparse/dense, small/large network) and we attempt to put some order into this diversity, motivated by the fact that many properties are qualitatively the same in all the models. Our main result is that random networks in all models give rise to cyclic behavior more often than fixed points. And although periodic orbits seem to dominate network dynamics, they are usually considered unstable and not allowed to survive in previous evolutionary studies. Defining stability as the probability of fixed points, we show that the stability distribution of these networks is highly robust to changes in its parameters. We also find sparser networks to be more stable, which may help to explain why they seem to be favored by evolution. We have unified several disconnected previous studies of this class of models under the framework of stability, in a way that had not been systematically explored before.

## Introduction

Gene regulatory networks have been studied intensively in recent years, both by physicists and biologists, who have provided different insights into this important field [1], [2]. We present numerical simulations to investigate stability in large Random Threshold Networks (RTNs) [3]. This spin glass or neural network-type model [4] represents a subclass of Random Boolean Networks (RBNs) [5]. We consider all attractor types rather than only those networks that have fixed points. Thus our framework is that used by physicists [6], [7] rather than that used by biologists [8], [9].

Wagner [8], [10] introduced a version of this gene network model to study the evolution of genetic robustness. The gene network is represented by a dynamical system whose state variables correspond to expression levels of the network's genes. A network is said to be robust if it retains the same expression state after mutation. The transient from an initial state to an attractor represents a developmental process and the fixed point attractor represents the phenotype. For this reason, fixed points have been traditionally considered developmentally stable, and networks that have cycling dynamics are not allowed to survive. This requirement of developmental stability [9] can be viewed as viability selection [11].

Implementations of the model for evolutionary simulations have varied in parameters such as network size, connectivity, normalization function, and whether the components of both the state vector and the matrix are discrete or continuous [8], [9], [11]–[22]. In principle, all of these parameters may influence the dynamics of the model and, consequently, the results of evolutionary simulations. It is well known, for example, that prior to evolution, smaller networks are more robust to mutations than larger ones, and that this relationship reverses after selection [8]. Our goal is to systematically explore how changes in all of these parameters affect the probability of fixed points, in the hope of motivating discussion of the relevance of this model for evolutionary analysis.

To this end,we focus our attention solely on the gene network model itself, on what has been called developmental dynamics, without evolution. We generate millions of random networks of size up to 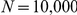 and measure the probability of fixed point dynamics for most of the different parameterizations reported in the literature, and show that cycles always dominate network dynamics. Fixed point steady states are the exception, not the rule in this gene network model. We also show that stability, defined as the probability of fixed point dynamics, decreases with network size and density. Stability distributions are bimodal: some matrices are always stable independently of the initial state, while others never reach a fixed point. Other measurements like period-size distributions show further deviation of the properties of this network model from those of the general class of RBNs.

The layout of the paper is the following: we finish the Introduction by presenting a more detailed version of the Model; we next present our Results (more of which are detailed in Text S2 and Supporting Information Figures), followed by a Discussion; we conclude with a short Methods section where we present a summary of each model variant used to produce our Figures (Table S1; more detailed methods can be found in Text S1).

### Model

The model consists of an interaction network of 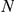 genes, represented by an 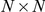 matrix, 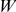, whose elements, 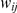, denote the effect on gene 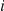 of the product of gene 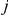. The matrix is generally not symmetric and diagonal elements, 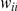, represent self-regulation. The fraction 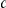 of nonzero entries in the 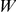 matrix is a parameter of the model and reflects the density of the network. The degree of a gene is represented by 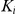 and 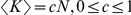 is called network connectivity [23], [24]. Each network is a dynamical system, with state vector 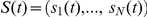 representing the expression levels of each gene at time 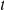. The deterministic, discrete-time dynamics of 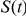 are modeled by the set of nonlinear coupled difference equations
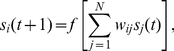
(1)where 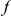 is a normalization function that prevents the system from diverging (Text S1). More specifically, 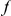 is a threshold function (either step or sigmoid; Figure S13), representing cooperative binding and saturation in gene expression. The network is updated synchronously (see [25]–[27] for asynchronous updates). We define Equation (1) as the development process (see [8], [9] for an illustration of the model, as well as a discussion of the biological motivations and assumptions behind it). Since the state space of the model is finite and the dynamics deterministic, the system will eventually reach an attractor given an initial gene expression state. The attractor can either be a fixed point or a limit cycle.

The simplicity of the model allows for evolutionary simulations, where a standard population genetic model of mutation, recombination and selection acts upon a population of gene networks, and the network's state is taken as its phenotype. Despite their level of abstraction, Boolean networks have been highly successful, both at reproducing experimental results in different organisms [2], [18], [28], [29], and allowing for theoretical predictions about the evolution of network properties such as robustness, evolvability, and many others [8], [9], [11], [13]–[17], [19]–[22], [30]–[38].

The properties of this gene network model in the absence of evolution or any kind of selection have attracted considerably less attention in the genetic regulation literature [11], [12], [18], [20], [30], [31]. On the other hand, the physics community has been studying some theoretical properties of RBNs for some time (see [6], [7] for recent reviews). In RBNS, each node is assigned an update function that prescribes the state of the node in the next time step, given the state of its input nodes. This update function is chosen from the set of all possible update functions according to some probability distribution. Since each of the 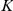 inputs of a node can be on or off, there are 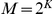 possible input states. The update function has to specify the new state of a node for each of these input states. Consequently, there are 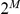 different update functions [7]. RTNs are boolean networks with threshold functions only. The update function is Equation (1) with 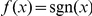 (Text S1).

While some analytical results have been obtained for the general class of RBNs, they usually apply only under some restricted conditions, such as in the limit of very large networks, specific network connectivities, or combinations of boolean functions. It has been shown that some results derived under these assumptions break down when only a subset of boolean functions is considered [39]–[41]. This is the case for RTNs, and although interesting in their own right, theoretical work done with RTNs seems to have been limited [3], [24], [42], [43].

## Results

**Figure 1:**
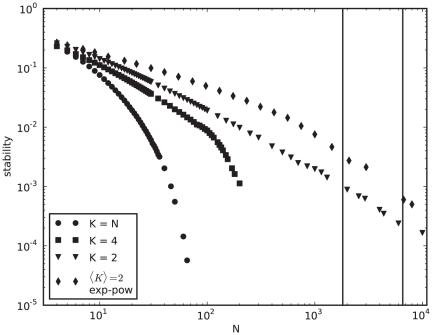
Cycles dominate the dynamics. Average stability (Equation (2)) for different network sizes 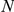, degree 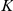 and topology. 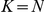 means 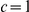. Equation (1) is solved up to 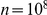 times with 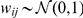, 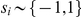, 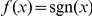 and 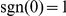. Noisy tails for 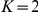 result from insufficient samples (Figure S4). Our measure is binary (the outcome is either 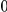 or 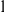 for each trial) and, for that reason, we do not find it helpful to present a variance measure. Instead, we present full stability distributions for similar experiments in Figure 2. Boxed region represents the size of the genome-wide regulatory networks of *E. coli* [45] and yeast [47].

As we can see in Figure 1, cycles seem to dominate the dynamics, independently of network size 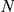 and degree 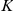. Stability (Equation (2) in Methods) has a maximum of 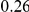 for 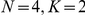, and decreases monotonically in both parameters. For 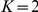, stability decreases almost as a power-law in 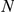, and this decrease is faster for larger 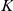. A minimal stability value of 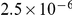 is found for 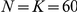. Small networks of 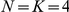 genes are about 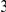 times more likely to reach a cycle than a fixed point steady state. For 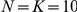, cycles are 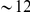 times more likely.

Also represented is a non-regular biological topology, with exponential in-degree distribution, and scale-free out-degrees (Text S1, Text S2, Figure S1 and Figure S2; see Table S2 and Figure S3 for transient times and Figure S4 for sample sizes).

**Figure 2:**
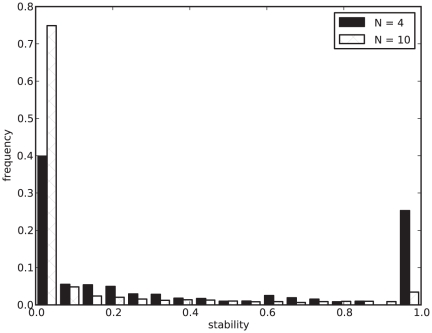
Stability distribution is bimodal. Two types of matrices: stable (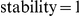) and unstable (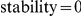). Full enumeration of binary network space for 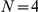 
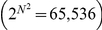
 and a random sample of 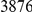 binary matrices for 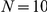. Full enumeration of the state space for both cases 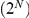. For 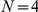, each bin corresponds to a specific stability value. There are a total of 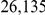 unstable (first bin) and 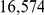 stable (last bin) matrices in the genotype space of binary matrices of size 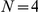. For 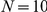, different stability values are binned together in 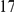 bins of width 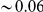 each. For that reason, not all 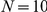 matrices included in the first bin have 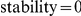, for example. There are 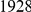 unstable matrices versus 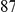 stable ones in our random sample of 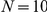. 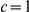; other parameters are as in Figure 1.

Figure 1 depicts the average stability for networks of different sizes. We next ask if this average is a good proxy for typical network behavior. In other words, what is the stability distribution for networks of a given size? Figure 2 shows that this distribution is bimodal, where some matrices are never stable, independently of the initial state, while others always reach a fixed point from every initial state. This suggests there could be two type of matrices in this model: unstable and stable ones, with the former being much more common than the latter. Interestingly, if we increase network size to 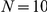 and sample random binary matrices, we still find both types of matrices, but with a more uneven distribution. We find 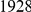 matrices with 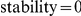 in our random sample of 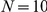, against 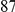 with 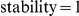: a 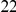-fold difference.

**Figure 3:**
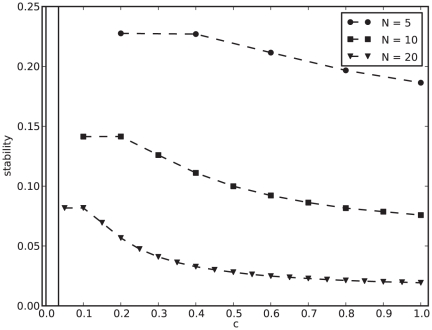
Sparser networks are more stable. Average stability (Equation (2)) for different network densities 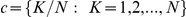 and sizes 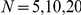. Equation (1) is solved up to 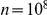 times for each 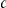 and 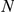. Other parameters as in Figure 1. Dashed lines are guides to the eye. Shaded region represents the density of biological networks [32], [45].

We next ask how network density, 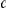, affects stability. As we can see in Figure 3, stability goes down with increasing 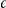, and has a maximum value of 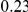 for 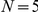 and minimal 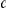. Again, cycles dominate the dynamics, independent of network density, but sparser networks seem more stable. Although sparse networks of size 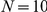 with one or two regulatory inputs per gene (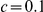 and 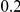) only have stability 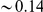, they are almost twice as stable as dense networks with 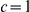.

**Figure 4:**
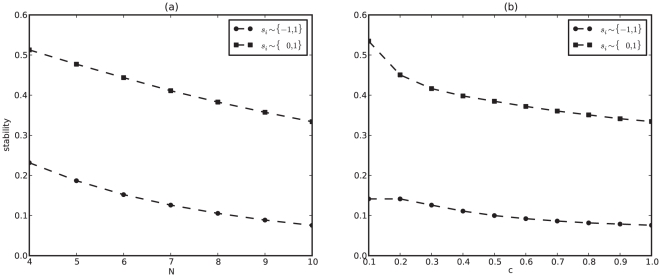
Stability is 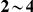
 **fold higher with the** 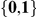 **(squares) than the** 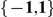 **(circles) maps.** Equation (1) is solved 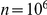 times for each 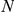, 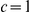 (a) and 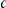, 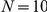 (b) with 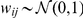. For the 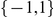 map: 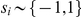, 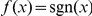, 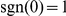. For the 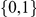 map 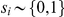, 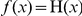, 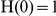 (Text S1). Dashed lines are guides to the eye.

So far we have represented the off state of a gene by 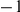, and although this seems to be the most common choice in the literature, some authors use 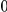 to represent the off state [18], [19]. As shown in Figure 4, stability is higher in the 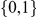 than the 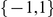 map, and it goes down linearly with both 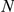 and 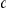. The slope of this decay is about the same for both maps, resulting in a 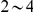 fold difference in stability between the two. Interestingly, the 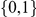 map produces the only instances for which reaching a fixed point is actually more likely than a cycle. This is the case for 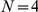, 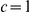 and 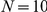, 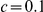. In fact, using the 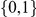 map, stability is always greater than 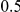 for the sparsest 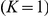 network of any size (Figure S6; see Text S2, Figure S7 and Figure S8 for more comparative studies of the two representations).

**Figure 5:**
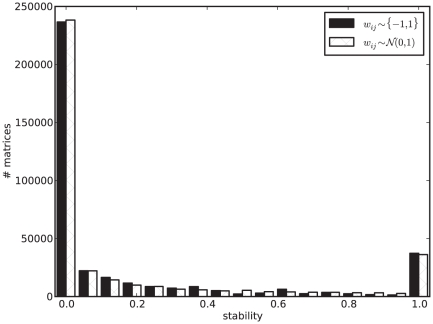
Binary and real matrices seem to have the same stability distribution. Equation (1) is solved for 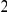 sets of 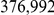 random matrices each, and full enumeration of state space 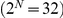. 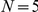, 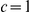, 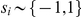, 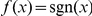, 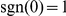. 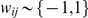 for binary matrices and 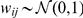 for real ones.

In Figure 5 we show that stability distributions are very similar for binary and real matrices, with the real set having slightly more unstable and fewer stable matrices than the binary one. To see whether the same is true of the normalized means of the distributions, i.e., the networks' average stability, we return to Figure 1 and see that for 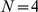 we find a stability value around 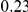, estimated by randomly sampling 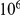 real matrices. Random sampling of 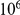 binary matrices estimates stability around 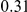. A full enumeration of the total 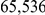 binary matrices (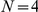) yields 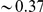 stability. It seems that random sampling over-represents unstable matrices, which are the most frequent ones in the full distribution.

**Figure 6:**
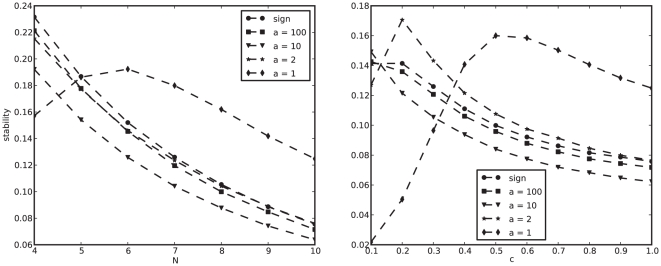
Steep sigmoid functions result in the same stability profiles as the sign function. Average stability (2) for different network sizes 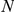, 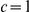 (a) and densities 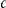, 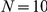 (b) with different normalization functions 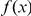. Equation (1) is solved 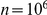 times with 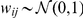, 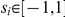, 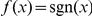 with 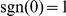 for the first curve, and the sigmoid 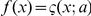 for all curves identified by steepness 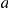 (Text S1). For 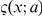, other parameters are 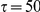, 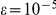, 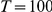 and 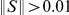. Dashed lines are guides to the eye.

Finally, we compare stability for binary and real states. Figure 6 shows that the stability distributions are similar with either the sign (binary) or the steep sigmoid (real) functions with 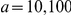 and even 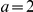 for all 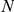 and 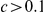. This is somewhat expected if you compare these different normalization functions in Figure S13. In fact, 
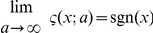
. We also note that stability starts to behave differently for low 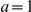 (Text S1, Text S2 and Figure S9).

More results are presented in Text S2.

## Discussion

In this study, we have conducted extensive simulation analysis of a subclass of random Boolean networks, known as Random Threshold Networks (RTNs). We defined stability as the probability of reaching a steady state and investigated the dependence of stability on network size and density, types of regulatory interactions and gene expressions and parameter values. The main findings are:

1. There are vastly more cyclic solutions than steady state solutions; only the latter have been assumed to be “viable” networks in previous studies.
2. Some networks never cycle while others always cycle independently of the initial conditions.
3. Sparse networks are stable more often than dense networks.
4. Using 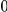 instead of 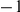 to represent the off state induces more stable solutions. However, for well connected networks, stable states are discovered more rapidly for 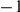 networks.
5. Binary and real valued weight matrices have similar stability properties.
6. Discrete or continuous gene expression states may give similar results, depending on the steepness of the sigmoid function.
7. Network topology seems to have a small effect on stability
8. The distribution of attractor lengths appears to decay more slowly than the typical power law.

All of these results may have implications for both the use of the RTN as a model of gene regulation and the properties of real biological networks. For brevity, we focus solely on the former.

This is the first time networks of the size of an organism have been simulated with this model. To the best of our knowledge, the largest network previously studied has 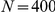 genes [44]. We have extended this range to 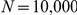 (Figure 1). This is comparable to *E. coli* (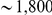 genes identified in its regulatory network [45] or 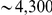 annotated genes in the genome of the K-12 strain [46]) and to yeast (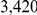 network genes [47] or 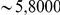 annotated genes in the *Saccharomyces cerevisiae* genome [48]). We have also seen that stability seems to decay much faster for degree 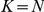 than for smaller 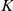. For networks of size 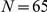, for example, the probability of fixed points is already smaller than 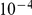. More importantly, this number seems to approach 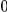 for larger 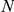, unlike the fat tails of 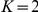. In other words, by choosing 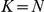, one cannot study stable states for organism-size, genome-wide networks. Interestingly, dense networks are the most popular choice in the literature [16], [22]. Our results have clear implications for the interpretation of previous studies [8], [16]. By limiting their analysis to small dense networks with stable dynamics, most previous findings are limited in reach and may not apply to real biological networks.

Figure 2 clearly suggests there are two types of networks in this model: stable and unstable. This bimodal nature of the stability distribution is not trivial. It is interesting that the stability of a random pair of matrix and initial state tells us how likely it is that the matrix is stable (or not) with any other random initial state. It is the network that determines stability, not the initial state. This implies that future studies using this model do not have to sample many different initial states to characterize network dynamics. Sampling pairs of networks and initial states, as we have done in most of our study, should suffice. These two classes of networks may have different topological properties. Since previous studies only use viable networks, most of the networks they allow to evolve are of the second type, i.e., stable. It is true that we do not expect biological networks to be random; the question is if we do want to start our simulations from a non-random set, are the chosen matrices biologically relevant? And, by selecting these and not others, what properties or biases are introduced into the simulations, prior to evolution? To the best of our knowledge, these questions have yet to be addressed.

A great deal has been written about scale-free networks in biology [49], [50]. In contrast, most networks we have studied here are regular, since this is the topology frequently used with gene regulatory networks [8], [9], and thus the ones we are interested in characterizing. We have shown, however, that different topologies, including scale-free out-degree distributions, do not seem to change the overall results, in agreement with previous studies [22], [34].

In Figure 3 we see that stability seems to decay faster with 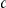 for larger networks and in Figure S5 we have tried to show this explicitly finding that for small to intermediate size networks (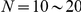), the difference between the stability of dense and sparse networks is maximized. This is about the size of networks used in previous studies [11], [33], where networks with different densities coexist in a population (i.e. adding or deleting connections is allowed). We believe this stability difference should be taken into account in analysis of such studies. Further, network stability does not seem to depend strongly on the nature of the matrix weights, either binary or real-valued (Figure 5).

An important parameter of the model is how cells respond to their input signals. That is to say, is gene regulation a switch-like process, or is the response graded? While the former is implemented by a step function, the latter is modeled as a sigmoid curve (Figure S13). One could argue that a switch-like mechanism already introduces a lot of robustness to the model, in the sense that most changes do not produce a visible change in the phenotype (for 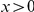 or 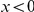 in the figure). Sensitivity sharply increases, however, at 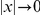, where the discontinuity occurs. Close to zero, very small changes in the regulatory inputs of a gene can quickly turn it on or off. In this 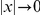 region, the system is clearly not robust. The opposite can be said of a not very steep sigmoid function. By allowing continuous expression values, small changes in 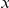 lead to small changes in a gene's state 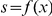. This choice, however, allows changes in positive or negative inputs around 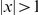 (or even 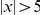) to still produce visible changes in phenotype. In this sense, the system is less robust. We have shown that the two choices are only equivalent, at least in terms of stability, for large 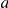, where the sigmoid behaves almost like a switch (Figure 6). With small 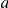, for which the sigmoid is less steep and behaves more like a gradient, the choice between a step [8], [14] or a sigmoid function [9], [16] makes a difference (see Text S2 and Figure S12 on how to deal with 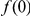 in the discrete case).

As already mentioned, a lot of work has been done on analytical properties of Random Boolean Networks [23], [51] and it has been suggested that some properties of RTNs do not follow analytical results derived for the general class of RBNs [39]–[41]. The dynamics of RBNs with canalizing functions only, for example, seem to be dominated by fixed points [52]. We have shown here that this is clearly not the case for RTNs. It has also been suggested that the attractor length distribution of RBNs follows a power-law [7], [40]. Again we have shown this is not true for RTNs, which instead seem to follow an exponential decay for a range of parameters (Figure S10 and Figure S11) perhaps slightly slower than initially suggested [43].

## Methods

The attractor reached by the dynamical system (1) is uniquely defined by the matrix 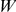 and the initial state 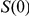, and is either a fixed point or a cycle. Let 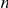 represent the number of pairs of 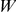 and 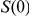 for which Equation (1) is solved, and 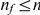 the number of times the attractor is a fixed point. To estimate the probability of reaching a fixed point steady state within the framework of this model, we generated up to 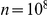 random pairs of matrices and initial states, for different network sizes 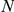 and degrees 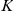, and measured
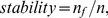
(2)which takes values between 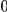 and 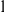. The estimated probability of cycles is given by 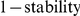.

As mentioned before, most of the evolutionary studies done with this model vary in the parameters used in Equation (1). We estimate the dependence of stability measured by Equation (2) on most of these parameters. We list in Table S1 all the variations we have studied along with the corresponding figures and references. Special relevance is given to the range of parameters used in previous studies. For this reason, we mostly study small [8], [9] and dense [16], [22] networks. Real gene networks, however, can be quite large [45], [47] and appear to be sparsely connected, with an average of two transcriptional regulators per gene [32]. Stability estimates are also shown for these more realistic topologies [49], [53].

More detailed methods and algorithms are included in Text S1.

## Supporting Information

Text S1 **Supporting Methods.** More details on experimental procedures and algorithms for solving Equation (1) for both the discrete and continuous cases.
(PDF)

Text S2 **Supporting Results.** More results exploring the dependence of stability on parameters of the model. More parameters are also explored. Sample sizes and transient times are analyzed too.
(PDF)

Figure S1
**Stability decreases with** 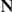 **even for scale-free biological topologies.** Average stability (Equation (2)) is plotted for different network sizes 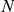 and topology. 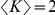. 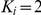 for every gene, 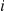, in the regular network. The Poisson network draws the degree distributions from a Poisson distribution with mean and variance equal to 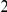. *exp-pow* stands for exponential in-degree distribution, and power-law out-degree distribution, both with mean 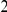. Other parameters as in Figure 1.(EPS)

Figure S2
**Stability decreases with** 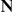 **in spite of topology for** 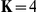 **.** Same as Figure S1 for 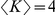. In this case we do not represent the biological topology, since biological networks usually have 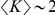 [32].(EPS)

Figure S3
**Path length to equilibrium grows rapidly with** 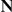 **.** Represented is average transient time (i.e. the number of time steps until the network reaches an attractor) as a function of network size 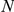 and for different degrees 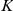. Only fixed points are considered. Equation (1) is solved up to 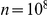 times with parameters as in Figure 1. Although coefficients of a least-squares fit are shown in the figure legend for each 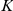, these regression lines are presented here only as a qualitative description. The estimates are employed for the extrapolation of transient times of larger networks, used to impose a cut-off on the convergence time of Equation (1). Error bars are big but are not shown to avoid cluttering the figure.(EPS)

Figure S4
**Sample size decreases with increasing** 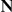 **.** Shown is the number of samples from which the results presented in Figure 1 are drawn. Increase of convergence time with 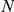, as depicted in Figure S3, limits sample size.(EPS)

Figure S5
**The effect of network degree on stability seems to depend on network size.** Represented is 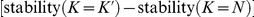 for different 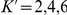 and 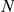. It seems that the difference in stability between sparse networks and the densest one has a maximum for an intermediate 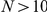. After an initial increase, it goes down with increasing 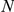, where 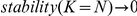, and thus the difference is reduced to 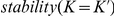. This basically represents the difference between the different plots in Figure 1.(EPS)

Figure S6
**Stability is higher than** 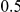 **for the** 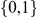 **map with** 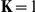 **.** The 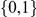 map produces the only case where reaching a fixed point is actually more likely than reaching a cycle for any network size. This happens, however, for the uninteresting case of 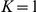 regular networks, where each gene receives input from only one other gene, or itself. Equation (1) is solved up to 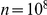 times for each 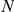 and 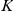 with 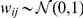, 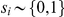, 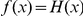 and 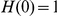 (Text S1).(EPS)

Figure S7
**The** 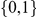 **map has more stable and less unstable matrices than** 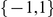 **.** Equation (1) is solved for 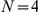, 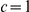, 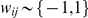 and full enumeration of the network 
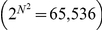
 and state 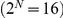 spaces. Other parameters are as in Figure 4.(EPS)

Figure S8
**The** 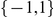 **map and real matrices allow for faster discovery of novel phenotypes.** Shown is the number of samples needed to reach all 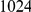 fixed point attractors of networks of size 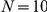, for different 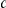 and maps (a) or types of regulatory interactions (either binary or real) (b). Equation (1) is solved up to 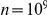 times for each 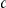. For 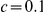 and 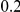, the 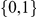 map reaches the maximum sample size before the discovery of all stable phenotypes. The results shown are for one run only. Other parameters are as in Figure 4 (a) and Figure 5 (b).(EPS)

Figure S9
**Stability is not monotonic in** 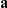 **.** Although still low, the probability of fixed points has a maximum for 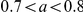. Stability also seems slightly higher for 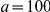 than 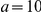. Equation (1) is solved 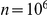 times with 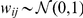, 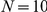, 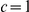, 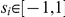 and sigmoidal 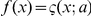, varying 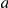 (Text S1). Other sigmoid parameters are as in Figure 6. The dashed line is a guide to the eye.(EPS)

Figure S10
**Probability of attractor length decays slower than a power-law.** Shown is the attractor period distribution for the two different maps and networks of size and density 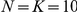. The 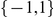 map has an antisymmetric property where cycles of even length are overrepresented at least 2-fold (Figure S11; [12], [31]). For that reason, even and odd periods are analyzed separately. Also shown is a least-squares fit for the 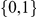 map. Equation (1) is solved with parameters as in Figure 4.(EPS)

Figure S11
**Cycle size distribution for the** 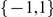 **map.** Represented is the attractor size distribution, as in Figure S10, but just for the 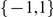 map with the odd and even length cycles taken together. Note how cycles of even length are overrepresented at least 2-fold.(EPS)

Figure S12
**Different conventions for** 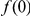 **result in similar stability profiles.** Stability is shown for different 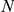 and definitions of 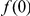 (Equation (1)). Networks are regular and binary, 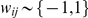. *random* means we choose 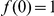 or 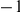 with equal probability. 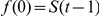 means 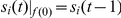. 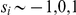 for the latter and also for 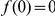.(EPS)

Figure S13
**The sign and sigmoid functions are very similar for** 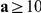 **.** The parameter 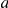 controls the steepness of the sigmoid function, 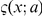, where 
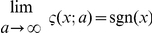
 (Text S1).(EPS)

Table S1 List of all model variants and corresponding figures and references. Most of the evolutionary studies done with this model vary in the parameters used in Equation (1). We estimate the dependence of stability measured by Equation (2) on most of these parameters.
(PDF)

Table S2Limits on transient times as a function of 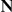 and 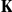. Table entries are the range of 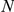 values for which each 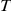 is used. The time it takes for Equation (1) to reach an attractor grows with 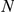 (Figure S3). To be able to produce Figure 1, a time limit 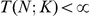 is enforced for large or dense networks.(PDF)
