# Supplementary material for: Most Networks in Wagner's Model Are Cycling
Source: PLoS One. 2012 Apr 12;7(4):e34285. doi: 10.1371/journal.pone.0034285 (PMC3325246; doi:10.1371/journal.pone.0034285)
Supplement: Text S1 — Supporting Methods. More details on experimental procedures and algorithms for solving Equation (1) for both the discrete and continuous cases. (PDF) [file pone.0034285.s001.pdf]

Text S1. Supporting Methods

Discrete dynamics

We either choose f in Equation (1) as the sign function sgn: $f(x) = -1$ if $x < 0$, $f(x) = 1$ if $x \geq 0$ or the Heaviside function H: $f(x) = 0$ if $x < 0$, $f(x) = 1$ if $x \geq 0$. $s_i(t)$ only takes the values -1 (in the former) or 0 (in the latter) representing the not expressed state, or 1 as the expressed state. This results in a dynamical system for $S(t)$ on the finite sets $\{-1, 1\}^N$ or $\{0, 1\}^N$.

Solving the dynamic equations

Since the N-dimensional state vector $S(t)$ is binary, we can represent it by an integer $x_i \in \{0, 1, ..., 2^N - 1\}$, using binary or gray code [54], for example. The matrix multiplication defined in Equation (1), however, can only be performed with $S(t)$ in the vector representation. But once normalized by f, we can map $S(t)$ to the integer representation. To solve Equation (1), we alternate between the two representations.

With $S(t)$ in the integer representation x_i, we can rewrite Equation (1) as $x_{i+1} = \mathcal{F}(x_i)$. $\mathcal{F}$ maps the finite set $\{0, 1, ..., 2^N - 1\}$ into itself deterministically, and starting from any initial value x_0, the sequence of iterated values x_0, $x_1 = \mathcal{F}(x_0)$, $x_2 = \mathcal{F}(x_1)$, ..., $x_i = \mathcal{F}(x_{i-1})$, ... must eventually use the same value twice: there must be some $i \neq j$ such that $x_i = x_j$. Once this happens, the sequence must continue by repeating the cycle of values from x_i to x_{j-1}. The problem of finding the solutions of Equation (1) can thus be solved by a cycle detection algorithm. We use Brent's algorithm [55] which returns the orbit's period (period of 1 represents a fixed point), the path length to equilibrium (transient time from the initial state to the attractor) and the final state of the system (or the first state of a cycle). With these elements in hand the full orbit can also be reconstructed.

Continuous dynamics

Throughout this study we have considered only discrete state spaces. Some authors, however, used continuously-valued state vectors [9, 16]. This is the case when the state vectors of Equation (1) are normalized with a sigmoid function. Following [9] we define

$$\varsigma(x; a) = \frac{2}{1 + e^{-ax}} - 1 \tag{3}$$

where the parameter a controls the steepness of the sigmoid - the larger a the steeper the function (Figure S13).

Solving the dynamic equations

When using $\varsigma(x; a)$ (Equation (3)), the equilibrium steady state, $S(\infty)$, is reached when a measure analogous to a variance,

$$\Psi\left(S(t)\right) = \frac{1}{\tau} \sum_{t'=t-\tau}^{t} d\big(S(t'), \bar{S}(t)\big), \tag{4}$$

is smaller than an error threshold $\epsilon \ll 1$, where $d(S^a, S^b) = \sum_{i=1}^{N} (s_i^a - s_i^b)^2 / N$ defines our distance metric between two state vectors S^a and S^b, and where $\bar{S}(t)$ is the average of states in the time interval $(t - \tau, ..., t)$. When this convergence criterion is satisfied within $T = 100$ iterations [9], a fixed point steady state is found such that $S(\infty) = \bar{S}(t)$. Usually $\tau = 10$ and $\epsilon = 10^{-4}$ [9].

Full enumeration of state space and stability distributions

For matrices of size $N = 4$ with binary elements $w_{ij} \sim \{-1, 1\}$, it is possible to fully enumerate both the network and the state spaces. For each network we solve Equation (1) starting from every possible initial state, and count how many times a fixed point is reached. The stability of a given network i, $1 \leq i \leq 2^{N^2}$ is given by stability$^{(i)} = n_f^{(i)}/n$, $n = 16$. A fixed point could be reached for none of the initial states ($n_f = 0$, stability $= 0$), for all initial states ($n_f = 16$, stability $= 1$), or for some intermediate number.

Network density, connectivity and topology

For comparison with relevant work [8,9], we use c in Figure 3 as a proxy for network connectivity, instead of K, which is most commonly used in the physics literature [5, 56]. We should note that the networks in this figure are regular and thus every gene has the same degree K given by $K = cN$, and that c takes only discrete values between 0 and 1.

Representing the off-state of a gene

The off state of a gene can either be represented by -1 (most commonly) or by 0 [18,19]. Huerta-Sanchez & Durrett [12] argue that 0 would be a more realistic choice since an expression state of -1 still has an effect on the expression of other genes. By comparing the two representations, they found the $\{0, 1\}$ mapping to have more fixed points than $\{-1, 1\}$ for fully connected ($c = 1$) networks of sizes $N = 4, 7, 10$. To generalize their results to different values of c and N, we redo the experiments pictured in Figure 1 and Figure 3 for the $\{0, 1\}$ map and plot them in Figure 4.

Binary vs. real matrices

So far we have chosen to use either binary $\{-1, 1\}$ or real $\mathcal{N}(0, 1)$ matrices, and we have done so rather arbitrarily. Although real matrices are the most common choice in the literature, some authors [11, 14] have chosen to use binary matrices. Computationally, however, the two approaches are quite different, with the space of binary matrices constituting a finite set, thus allowing its full enumeration for small networks ($N \leq 5$, $c = 1$). To show that the reported stability measures are, to a good approximation, independent of this choice, we generated two sets of 376, 992 random matrices each, one sampled from the class of binary matrices and the other from real numbers, all of network size $N = 5$. For each matrix we solve Equation (1) for all 32 initial states and count the number of times it reaches a fixed point. The resulting stability distributions, analogous to those in Figure 2, are plotted in Figure 5.

Developmental or transient time

The time it takes for Equation (1) to reach an attractor grows with N (Figure S3). Using small networks of $N = 10$, previous authors [8,9] have put a limit on the number of iterations, $T = 100$, of Equation (1), until it converges according to the criterion defined in Equation (4). If a fixed point has not been reached after T iterations, the network is said unstable for the given initial state. By using Brent's algorithm [55] for discrete dynamics in a highly optimized code, we usually allow the system to iterate until a fixed point or a cycle has been found ($T = \infty$). But to be able to produce Figure 1, a limit $T(N; K) < \infty$ is enforced for large or dense networks (Table S2). We use two limits. The first one is based on the longest convergence time observed for any network of a given size and degree, $T(N; K)_{max}$. The second on it's average, $T(N; K)_{mean}$. The latter is estimated from the regression lines for $T = \infty$ shown in Figure S3, and extrapolated to larger N. The same holds for $T(N; K)_{max}$. Both are incremented in powers of two, according to the implementation of Brent's algorithm [55].
