# Supplementary material for: Most Networks in Wagner's Model Are Cycling
Source: PLoS One. 2012 Apr 12;7(4):e34285. doi: 10.1371/journal.pone.0034285 (PMC3325246; doi:10.1371/journal.pone.0034285)
Supplement: Text S2 — Supporting Results. More results exploring the dependence of stability on parameters of the model. More parameters are also explored. Sample sizes and transient times are analyzed too. (PDF) [file pone.0034285.s002.pdf]

Text S2. Supporting Results

Stability decreases with N in spite of topology

Throughout this paper we use regular networks, where every gene, i, has the same degree, $K_i = \langle K \rangle$. Biological networks, however, are far from regular [49, 53]. To see how different network topologies modify the results presented in Figure 1, we estimate stability in Figure S1 and Figure S2 for regular and Poisson degree distributions, as well the exponential in-degree, power-law out-degree distributed biological network already presented in Figure 1, with average degree $avgK = 2$. It seems more complex topology increases stability. The Poisson distributed $avgK = 2$ network may have such higher stability due to most of the genes not having regularity inputs, and hence the choice $f(0) = 1$ fixes these genes on - see section Using different step functions. We can see that the difference in stability between the two topologies with $K = 4$ is lower, probably due to the fact that most genes have at least one regulatory input.

Path length to equilibrium grows rapidly with N

The time it takes for the system to converge to a fixed point seems to increase exponentially with N for $K = 4$ and $K = N$, and slightly faster than $\sqrt{N}$ for $K = 2$ (Figure S3). Higher K requires longer convergence times. For that reason, sampling is limited to $N = 65, 200$ and $10,000$ for $K = N, 4$ and 2 respectively, with larger N having smaller sample sizes (Figure S4).

The effect of network degree on stability seems to depend on network size

There seems to be a dependence on N in the rate at which stability decays with c (Figure 3). To make this explicit, we show in Figure S5 the difference in stability between sparse networks, with $c = K'/N$, $K' = 2, 4, 6$, and the densest, with $K = N$, as a function of N. We see that the effect of network density in stability increases with network size, up to a maximum level at intermediate N. After this maximum, stability($K = N$) goes to 0 with increasing N, which makes the plot [stability($K = K'$) − stability($K = N$)] decay simply as stability($K = K'$).

Binary matrices with $\{0, 1\}$ have higher stability

We have seen that binary matrices usually have slightly higher stability than real ones (Figure 5). We have also shown that the $\{0, 1\}$ map results in higher stability (Figure 4). Combining these two choices yields high stability values of $0.6 \sim 0.7$ for sparse binary networks of $K = 1$ and 2 of any size $4 \leq N \leq 10$ (results not shown).

We now fully enumerate both the genotypic and phenotypic spaces and plot the stability distribution of binary matrices of size $N = 4$ as in Figure 2, for both maps, and compare them in Figure S7. The bimodal distribution is conserved, with the $\{0, 1\}$ map having more stable and less unstable matrices than $\{-1, 1\}$ (note that there are $n = 17$ bins corresponding to all possible values of stability in this experiment).

We should also mention that reproducing Figure 3 with the $\{0, 1\}$ map is only possible for $N > 5$. For smaller N, stability is not monotonic in c (results not shown).

The $\{-1, 1\}$ map and real matrices allow for faster discovery of novel phenotypes

Another difference between representing non-expression by 0 or -1 arises when searching the state space for novel phenotypes. For $c < 0.8$, the $\{-1, 1\}$ map usually allows for faster discovery of new phenotypes (fixed points) than $\{0, 1\}$ (Figure S8.a).

It is also worth mentioning that random sampling of real matrices is usually faster in discovering new phenotypes (fixed points) than sampling of binary ones (Figure S8.b).

Binary vs. real states

In general, stability is lower for the continuous case, but, surprisingly, it is not monotonic in a (Figure S9). Interestingly, although intermediate values of $a \sim 5$ or 10 are less stable than $a = 100$, stability increases sharply for $a < 5$. It has its maximum around $0.7 < a < 0.8$, and it goes down to zero very fast for smaller a. For $a < 0.6$ it becomes necessary to require $\|S\| > 0.001$ when evaluating stability, due to the predominance of very small expression values, $s_i \ll 1$, that approach 0 with iteration of Equation (1). These very small values satisfy the convergence criterion defined in Equation (4), but should not be considered stable.

Our results are also generally robust to changes in the parameters of Equation (4). For $N = 10$, $c = 1$ and $a = 100$, for example, stability is nearly constant for any $\tau < T - 15$ and $\epsilon < 10^{-2}$ (results not shown). Sensitivity increases for small a, however. For example, for $a \sim 0.7$, different choices of these parameters may change stability values by more than 2-fold, but still keep the fraction of fixed points below 0.3, and usually much lower than that (results not shown).

Probability of attractor length decays slower than a power-law

As already mentioned, a lot of work has been done on analytical properties of Random Boolean Networks [23, 51]. In this general class of models, the *update function* that specifies the state of a node in the next time step, given the state of its K inputs at the present time step, can take forms that differ from Equation (1). Moreover, each node in the network is randomly assigned an update function according to some probability distribution. For this type of network with $K = N$, for example, where all possible update functions are chosen with the same probability, it is predicted that an attractor of length (period) l occurs with probability $1/l$ if $l \ll 2^{N/2}$ [7, 40]. From what we have seen so far, this does not seem to apply to the present model. For example, we have shown that for $N = K = 10$, cycles ($l > 1$) are ~ 12 times more likely than fixed points ($l = 1$). For the above formula to hold, it would require that we sum the probabilities of cycles up to a length of about 2.7×10^5, which is clearly above the limit $2^{N/2} = 32$ (which according to [7], is of the order of the length of the largest attractor). It seems that the probability of cycles may decay slower than predicted (see also [12, 31]). To show this explicitly, we plot in Figure S10 the attractor length distribution for networks of size and density $N = K = 10$ and for both the $\{0, 1\}$ and $\{-1, 1\}$ maps. Due to an antisymmetric property of the latter [12, 31], we plot the odd and even periods separately - see Figure S11 where we show them together in linear scale. We can also see in Figure S10 that the period distribution of the $\{0, 1\}$ map seems to follow an exponential decay (slower than the $\exp(-l)$ predicted in [43]).

Using different step functions

One of the reasons we choose to use regular matrices is to avoid all-zero rows in the matrix W. That would represent a gene without any regulatory inputs. According to the matrix multiplication defined in Equation (1), an all-zero row i results in $s_i(t) = f(0)$, independently of $s_i(t-1)$. Different step functions f have different definitions for $f(0)$, and different choices have been made in the literature. Wagner [8], for example, has used $f(0) = 0$. Li *et al.* [18] chose $s_i(t) \mid_{f(0)} = s_i(t-1)$. McDonald *et al.* [20] randomly chose $f(0) = 1 \vee -1$ with equal probability. To see how these different choices affect stability, we plot in Figure S12 stability against N for all these different choices, for binary regular dense networks. When using binary weights $-1, 1$ with equal probability, even N will, on average, yield $f(0)$. For this reason, each step function produces two curves: one for even N and another for odd N. We can see that $f(0) = 1$, $f(0) = -1$ and $f(0) = 1 \vee -1$ with equal probability, behave very similarly. The difference is in

the average number of genes that are on and those that are off (results not shown). However, when we allow a third gene expression state, 0, in addition to on and off, coded by 1 and -1 respectively, stability decreases for even N. We believe this happens because the total number of cycles in attractor space scale faster than the number of fixed points, with the available number of gene expression states (results not shown). For this reason, although the total number of possible fixed points increase from 2^N to 3^N, the total number of cycles increases much more, and hence stability decreases.
