# Supplementary material for: Most Networks in Wagner's Model Are Cycling
Source: PLoS One. 2012 Apr 12;7(4):e34285. doi: 10.1371/journal.pone.0034285 (PMC3325246; doi:10.1371/journal.pone.0034285)
Supplement: Table S1 — List of all model variants and corresponding figures and references. Most of the evolutionary studies done with this model vary in the parameters used in Equation (1). We estimate the dependence of stability measured by Equation (2) on most of these parameters. (PDF) [file pone.0034285.s016.pdf]

**Table S1**

| Figure | what? | N | K | topology | $w_{ij}$ | $s_i$ | $f(x)$ | $f(0)$ | references |
|---|---|---|---|---|---|---|---|---|---|
| 1 | network size | $4..10,000$ | $N,4,2$ | regular, exp-pow | real $\sim \mathcal{N}(0,1)$ | $\{-1,1\}$ | $\mathrm{sgn}(x)$ | 1 | [8,9] |
| 2 | initial state | $4,10$ | $N$ | regular | real $\sim \mathcal{N}(0,1)$, binary $\sim \{-1,1\}$ | $\{-1,1\}$ | $\mathrm{sgn}(x)$ | 1 | [11] |
| 3 | network density | $5,10,20$ | $N..1$ | regular | real $\sim \mathcal{N}(0,1)$ | $\{-1,1\}$ | $\mathrm{sgn}(x)$ | 1 | [5,32,35,54] |
| 4 | off-state 0 or $-1$ | $4..10$ | $N..1$ | regular | real $\sim \mathcal{N}(0,1)$ | $\{-1,1\}$, $\{0,1\}$ | $\mathrm{sgn}(x)$, $\mathrm{H}(x)$ | 1 | [12,18–20] |
| 5 | binary or real matrices | 5 | $N$ | regular | real $\sim \mathcal{N}(0,1)$, binary $\sim \{-1,1\}$ | $\{-1,1\}$ | $\mathrm{sgn}(x)$ | 1 | [11,14,15] |
| 6 | binary or real states | $4..10$ | $N..1$ | regular | real $\sim \mathcal{N}(0,1)$ | $\{-1,1\}$, $[-1,1]$ | $\mathrm{sgn}(x)$, $\varsigma(x;a)$ | 1, 0 | [9,16] |
| S1 | topology $K=2$ | $4..10,000$ | 2 | regular, exp-pow, Poisson | real $\sim \mathcal{N}(0,1)$ | $\{-1,1\}$ | $\mathrm{sgn}(x)$ | 1 | [49,53] |
| S2 | topology $K=4$ | $4..250$ | 4 | regular, Poisson | real $\sim \mathcal{N}(0,1)$ | $\{-1,1\}$ | $\mathrm{sgn}(x)$ | 1 | |
| S3 | transient time | $4..1,000$ | $N,4,2$ | regular | real $\sim \mathcal{N}(0,1)$ | $\{-1,1\}$ | $\mathrm{sgn}(x)$ | 1 | |
| S4 | sample size | $4..10,000$ | $N,4,2$ | regular | real $\sim \mathcal{N}(0,1)$ | $\{-1,1\}$ | $\mathrm{sgn}(x)$ | 1 | |
| S5 | size and density | $4..60$ | $N,6,4,2$ | regular | real $\sim \mathcal{N}(0,1)$ | $\{-1,1\}$ | $\mathrm{sgn}(x)$ | 1 | |
| S6 | stability $> .5$ | $4..1,000$ | $2,1$ | regular | real $\sim \mathcal{N}(0,1)$ | $\{0,1\}$ | $\mathrm{H}(x)$ | 1 | |
| S7 | off-state 0 or $-1$ | 4 | $N$ | regular | binary $\sim \{-1,1\}$ | $\{-1,1\}$, $\{0,1\}$ | $\mathrm{sgn}(x)$, $\mathrm{H}(x)$ | 1 | |
| S8 | discovery time | 10 | $N..1$ | regular | real $\sim \mathcal{N}(0,1)$, binary $\sim \{-1,1\}$ | $\{-1,1\}$, $\{0,1\}$ | $\mathrm{sgn}(x)$, $\mathrm{H}(x)$ | 1 | |
| S9 | Hill number | 10 | $N$ | regular | real $\sim \mathcal{N}(0,1)$ | $[-1,1]$ | $\varsigma(x;a)$ | 0 | [11,17] |
| S10 | attractor length | 10 | $N$ | regular | real $\sim \mathcal{N}(0,1)$ | $\{-1,1\}$, $\{0,1\}$ | $\mathrm{sgn}(x)$, $\mathrm{H}(x)$ | 1 | [7,23,40,43] |
| S11 | odd and even length | 10 | $N$ | regular | real $\sim \mathcal{N}(0,1)$ | $\{-1,1\}$ | $\mathrm{sgn}(x)$ | 1 | [5,12] |
| S12 | $f(0)$ | $4..60$ | $N$ | regular | binary $\sim \{-1,1\}$ | $\{-1,1\}$ | $\mathrm{sgn}(x)$ | $\pm1,0$ | [8,18,20] |
| S13 | $f(x)$ | | | | | | $\mathrm{sgn}(x)$, $\varsigma(x;a)$ | 1, 0 | [11,17] |
