# Supplementary material for: Most Networks in Wagner's Model Are Cycling
Source: PLoS One. 2012 Apr 12;7(4):e34285. doi: 10.1371/journal.pone.0034285 (PMC3325246; doi:10.1371/journal.pone.0034285)
Supplement: Table S2 — Limits on transient times as a function of and . Table entries are the range of values for which each is used. The time it takes for Equation (1) to reach an attractor grows with (Figure S3). To be able to produce Figure 1, a time limit is enforced for large or dense networks. (PDF) [file pone.0034285.s017.pdf]

Table S2

K	T_∞	T_{max}	T_{mean}
N	$4..40$	$45, 50$	$55, 60, 65$
4	$4..100$	$105..140$	$145..200$
2	$4..1,000$	$1,150..4,373$	$4,500..10,000$
$\langle K \rangle = 2$	$4..747$	$1,000..6,694$	$8,000$
